# Cost of illness in patients with post-treatment Lyme disease syndrome in Belgium

**DOI:** 10.1093/eurpub/ckad045

**Published:** 2023-03-27

**Authors:** Ruben Willems, Nick Verhaeghe, Christian Perronne, Liesbeth Borgermans, Lieven Annemans

**Affiliations:** Department of Public Health and Primary Care, Interuniversity Centre for Health Economics Research (I-CHER), Ghent University, Gent, Belgium; Department of Public Health and Primary Care, Interuniversity Centre for Health Economics Research (I-CHER), Ghent University, Gent, Belgium; Infectious Diseases Department, University Hospital Raymond Poincaré, APHP, Université de Versailles Saint-Quentin—Paris Saclay, Garches, France; Department of Public Health and Primary Care, Interuniversity Centre for Health Economics Research (I-CHER), Ghent University, Gent, Belgium; Department of Public Health and Primary Care, Interuniversity Centre for Health Economics Research (I-CHER), Ghent University, Gent, Belgium

## Abstract

**Background:**

A proportion of patients with Lyme borreliosis (LB) report long-term persisting signs and symptoms, even after recommended antibiotic treatment, which is termed post-treatment Lyme disease syndrome (PTLDS). Consensus on guidance regarding diagnosis and treatment is currently lacking. Consequently, patients suffer and are left searching for answers, negatively impacting their quality of life and healthcare expenditure. Yet, health economic data on PTLDS remain scarce. The aim of this article is therefore to assess the cost-of-illness related to PTLDS, including the patient perspective.

**Methods:**

PTLDS patients (*N* = 187) with confirmed diagnosis of LB were recruited by a patient organization. Patients completed a self-reported questionnaire on LB-related healthcare utilization, absence from work and unemployment. Unit costs (reference year 2018) were obtained from national databases and published literature. Mean costs and uncertainty intervals were calculated via bootstrapping. Data were extrapolated to the Belgian population. Generalized linear models were used to determine associated covariates with total direct costs and out-of-pocket expenditures.

**Results:**

Mean annual direct costs amounted to €4618 (95% CI €4070–5152), of which 49.5% were out-of-pocket expenditures. Mean annual indirect costs amounted to €36 081 (€31 312–40 923). Direct and indirect costs at the population level were estimated at €19.4 and 151.5 million, respectively. A sickness or disability benefit as source of income was associated with higher direct and out-of-pocket costs.

**Conclusions:**

The economic burden associated with PTLDS on patients and society is substantial, with patients consuming large amounts of non-reimbursed healthcare resources. Guidance on adequate diagnosis and treatment of PTLDS is needed.

## Introduction

Lyme borreliosis (LB) is caused by spirochetes of the *Borrelia burgdorferi* (Bb) genospecies complex sensu lato, and is transmitted by ticks.^1^ It is the most common vector-borne illness in Europe, with 65 000 to over 200 000 cases being reported annually.^2^^,^^3^ However, this range is a significant underestimate, as case reporting is highly inconsistent, and many infections are not diagnosed.^4^^,^^5^ Although more countries are reporting the burden of LB, few countries in Europe have designated LB as a compulsory notifiable disease.^6^

Erythema migrans (EM), which manifests as expanding gyrate erythema at the site of a tick bite, is the most common objective manifestation of *Bb* infection. Clinical manifestations of LB may be both cutaneous and systemic, and can have cardiovascular, neurological and musculoskeletal involvement.^7^^,^^8^ Long-term outcomes are good when Lyme disease is treated at the early stage with appropriate antibiotic therapy.^9^ However, 5–10% of patients on initial antibiotic therapy develop post-treatment Lyme disease syndrome (PTLDS).^10^ This syndrome is a constellation of persistent symptoms, including joint and bone pain, arthritis, fatigue, cognitive difficulties, radicular pain and encephalitis.^7^^,^^11^^,^^12^ These symptoms have a consequent significant impact on the quality of life of patients for more than 6 months after treatment.^13^ There is growing evidence that treatment-refractory infection is related to persistent Bb infection.^14–17^ This issue has been recognized by the US Department of Health and Human Services.^18^ Evaluation and characterization of persistent symptoms is further complicated by the potential for co-morbid diseases that have overlapping symptom profiles.^13^ Consensus on guidance regarding diagnosis and treatment of PTLDS is currently lacking. Consequently, patients suffer and are left searching for answers, negatively impacting their quality of life and healthcare expenditure.^19^

Few studies have analyzed the economic burden of Lyme disease in Europe.^20^ Cost estimates from the societal perspective have been reported for Sweden,^21^ the Netherlands^22^ and Germany.^23^ Existing data show that 23.1–48.2% of total costs are indirect non-medical costs (e.g. production loss due to work absenteeism). However, in the Netherlands during 2010, Lyme disease-related costs varied across infection severity groups, with total annual societal costs per patient adding up to €53, 122, 5666 and 5697 for a tick bite, EM, disseminated LB and persisting symptoms, respectively.^22^ A recently published Belgian study estimated €193 per patient with EM and €5148 per patient with disseminated LB.^24^

The same Belgian study included few patients with PTLSD and called for more research in this patient population.^24^ Hence, data on direct and indirect costs attributable to persistent symptoms are still lacking for Belgium. Information is also limited on possible non-reimbursable healthcare utilization, as such data are not included in administrative databases. Thus, this study estimated the direct and indirect costs of confirmed PTLDS patients in Belgium, thereby also focussing on out-of-pocket expenditure. A cross-sectional design using an online self-reporting questionnaire was applied to collect data. The results provide more insight in patients’ healthcare seeking behaviour in a reality where guidance regarding diagnosis and treatment of PTLDS is lacking.

## Methods

### Study design

The study was performed within a cost-of-illness framework.^25^ Direct and indirect costs associated with the management of PTLDS patients were measured in Belgium during 2018. A prevalence-based approach^26^ was used to assess the societal economic burden of PTLDS over this period.^27^

### Study population and recruitment

Convenience sampling was applied via the digital newsletter and Facebook page of a non-profit Lyme disease patient association. The digital survey was available in Dutch and French. The study population included individuals of all ages living in Belgium with PTLDS, defined as LB-related persistent signs, with symptoms for more than 1 year after initial symptoms. To be included, individuals with PTLDS had to have a confirmation of (previous) Lyme disease, based on a physician and/or laboratory blood sample testing.

### Data collection

Cross-sectional data collection was assimilated using an online self-reporting questionnaire. Self-reporting was chosen to capture all healthcare utilization, even reimbursed care, because of the limited use of administrative data in not well-defined patient populations, such as the PTLDS population at hand. Moreover, we aimed to identify LB-related healthcare utilization data only, which would have been difficult, if not impossible, using administrative claims data.

Questions were related to: (i) sociodemographic information (i.e. sex, age, marital status, education and type of income), (ii) disease characteristics (i.e. diagnosis, testing and disease status), (iii) healthcare resource utilization [i.e. general practitioner (GP) visits, medical specialist visits, medical examinations, home-based nursing care, hospital care, pharmaceuticals and complementary treatment] and (iv) impact on work situation (i.e. employment status and absenteeism). To limit recall bias, data collection on healthcare resource utilization covered a retrospective period of 6 months before the survey was completed, except for hospitalization, which covered a 1-year period. Employment-related questions also covered a 1-year period. The questionnaire was launched mid-September 2018 via Time for Lyme (a patient organization), and remained open until the end of October 2018. A reminder to participate was sent after 5 weeks. At the time of the survey, the patient organization had 260 members, 2510 followers on Facebook and 835 newsletter subscribers.

### Cost categories: data sources and calculation of costs

Direct medical costs were calculated by applying unit costs to different types of healthcare resource utilization. Unit costs were obtained from the publicly available Nomenclature of Medical Performances database of the National Institute for Health and Disability Insurance (NIHDI). These costs were related to GPs, medical specialists, paramedics (other than psychologists), imaging and home-based nursing care. This database contains unit costs of all (partly) reimbursable performance that is included in compulsory health insurance.^28^ Unit costs for visits to psychologists could not be derived from this source, because the NIHDI did not consider reimbursement for visits to psychologists in 2018. The cost of a visit to a psychologist was obtained from a published report on the organization and reimbursement of psychological and orthopedagogical care in Belgium.^29^ An out-of-pocket cost of €47.02 for a 45-min consultation was derived.

Hospitalization costs were based on the average costs for hospital stays at the all-patient diagnosis related groups (APR-DRG) level. These costs were obtained from the publicly available National Database of Medical Diagnosis, Care and Cost.^30^ Participants were asked to indicate the reason why they were hospitalized during the reference period. Subsequently, the National Database of Medical Diagnosis, Care and Cost was consulted to obtain the average cost at the APR-DRG level. For example, ‘intestinal infection’ was reported as a reason for hospitalization. For this item, we used the APR-DRG code 245 ‘inflammatory bowel diseases’.

Costs related to a day hospitalization (without an overnight stay) were obtained from a report on the financing of day hospitalization in Belgium.^31^ The unit costs for prescribed medication were derived from the publicly available Drug Repository database of the Belgian Centre for Pharmacotherapeutic Information.^32^ For non-prescription (or over-the-counter) pharmaceuticals (mainly vitamins, other nutritional supplements and herbal medicines), cost information was obtained from the product websites. The price/dose/day for each drug was calculated and multiplied by the number of days medication was used in the 6-month period. For complementary treatments (e.g. acupuncture and osteopathy), participants were asked to report the type of treatment, number of sessions in the 6-month period and cost per session.

All unit costs were reported separately by both the patient and a third party (public health insurance). NIHDI and the Drug Repository database stratify between out-of-pocket and reimbursed costs, but hospitalization and day hospitalization costs were not stratified. For the latter, we assumed an out-of-pocket share based on a report of the largest Belgian health insurer.^33^ For all direct cost categories, except ‘day hospitalization’ and ‘hospitalization’, healthcare resource use information referred to a 6-month period. Costs were multiplied by two to reflect the annual cost.

Indirect non-medical costs included productivity losses related to: (i) temporary absence from work during the 1-year reference period before the questionnaire was completed; and (ii) unemployment related to post-treatment Lyme Disease symptoms. The Human Capital Approach was applied to reflect a calculus expressing the full quantification of lost economic productivity, including the consequences of losing employment as a result of the condition. For temporary absence from work, costs were estimated by multiplying the reported number of days of absence from work by an average unit cost of €284/day^34^ to not discriminate between people based on sociodemographic characteristics, such as age or sex.^35^ For people being unemployed for at least 1 year, 240 unemployment days per individual were applied (52 weeks in a year with 5 working days per week makes 260 working days, minus a legal minimum 20 days leave). For unemployment that started during the reference period, 120 unemployment days per individual were estimated, assuming that unemployment occurred in the middle of the year on average.

All costs were converted to and reported in Euros based on 2018 values.

### Data analysis

Data were analyzed with SPSS 27.0 software. A *P*-value of ≤0.05 was considered statistically significant. Standard summary statistics were used to describe participant characteristics. Categorical variables were presented as frequencies and percentages. Continuous variables were presented as means with standard deviations or medians with interquartile ranges (IQRs).

Mean direct medical costs were calculated for each category of healthcare utilization. 95% bias-corrected and accelerated confidence intervals were estimated using a bootstrap procedure with 1000 iterations. Missing data on healthcare utilization costs were interpolated using the mean value for a given category.

Generalized linear models with log-link function and gamma distribution (Tweedie distribution for indirect costs), reflecting the positively skewed distribution with high kurtosis and a heavy tail, were applied to investigate potential predictors of four dependent variables: (i) total direct costs, (ii) out-of-pocket costs of patients, (iii) total costs and (iv) indirect costs. Six covariates were included in the four separate models; gender, age, region, education, income type and time to diagnosis (defined as the time between first signs/onset of symptoms and the moment of diagnosis by testing and/or by a physician). The former five covariates were chosen because of the general likelihood that they would have an association with healthcare utilization and related costs. Time to diagnosis was chosen as a covariate because of its importance in patients with PTLDS. Multicollinearity diagnostics were performed, but none was detected. Participants with missing data on one of the covariates were excluded from regression analyses, which did not undermine model fit. Akaike Information Criterion appeared to be better when all six covariates were retained in the model, despite increasing the number of case-wise deletions, when compared to the scenario in which the covariate ‘time to diagnosis’ was excluded. Time to diagnosis accounted for the larger part of missing data.

Descriptive statistics were used to show the proportion of reimbursed versus out-of-pocket costs, as well as indirect non-medical costs.

### Extrapolation of findings to the population level

Numbers on the incidence of PTLDS are not available for Belgium. To extrapolate numbers for the Belgian population as a whole, we used data from the Netherlands,^22^ which reported an annual incidence figure of 905 cases for persisting signs and symptoms. This value was adjusted for Belgium by calculating the ratio of the number of EM and disseminated Lyme disease cases in Belgium to those for the Netherlands, producing a ratio of 0.49.^22^^,^^36^^,^^37^ This ratio was applied to the Netherlands incidence value (*n* = 905) to become an estimate of the number of persistent signs and symptoms cases in Belgium, being 447 per year. This incidence value was multiplied by a mean disease duration of 112.7 months (i.e. time between the first signs and symptoms of occurrence and Lyme diagnosis reported by participants at the time of filling out the questionnaire) resulting in a prevalence of 4199 patients (i.e. formula: prevalence=incidence × disease duration).

## Results

### Patient population characteristics

Characteristics of the 187 PTLDS patients who filled out the survey are provided in table 1. There was 1 missing data point on education, 1 missing data point on region and 55 missing data points on time to diagnosis. Most participants were females (80.2%), and were from Flanders (78.6%). The average age was 44.2 ± 12.9 years. Lyme disease was diagnosed in 88.8% of the study population based on blood sample testing (ELISA, Western Blot or LTT Elispot).

**Table 1:** Sociodemographic and disease characteristics of the study population (*n* = 187)

Variable	Study population
Gender, *n* (%)	
Male	37 (19.8)
Female	150 (80.2)
Age (years), mean ± SD	44.2 ± 12.9
Age categories (years), *n* (%)	
<40	68 (36.4)
40–59	96 (51.3)
≥60	23 (12.3)
Region	
Brussels	8 (4.3)
Flanders	147 (78.6)
Wallonia	31 (16.6)
Missing	1 (0.5)
Marital status, *n* (%)	
Married	68 (36.4)
Cohabiting	53 (28.3)
Single	46 (24.6)
Divorced	18 (9.6)
Widowed	2 (1.1)
Education, *n* (%)	
Primary school	10 (5.3)
Secondary school	45 (24.1)
University college	88 (47.1)
University	43 (23.0)
Missing	1 (0.5)
Income type, *n* (%)	
Disability benefit	69 (36.9)
Work	56 (29.9)
No source of income	17 (9.1)
Other^a^	45 (24.1)
Diagnosis, *n* (%)	
By blood sample testing	166 (88.8)
By physician’s opinion	21 (11.2)
Time from first symptoms to Lyme diagnosis	112.7 ± 113.0
Time to diagnosis categories (months), *n* (%)	
<12	26 (13.9)
12–60	34 (18.2)
61–120	32 (17.1)
≥120	40 (21.4)
Missing	55 (29.4)

a Other income types: pension, mixed work and disability benefit, living wage.

### Costs per category and extrapolation to the population level

**Figure 1:**
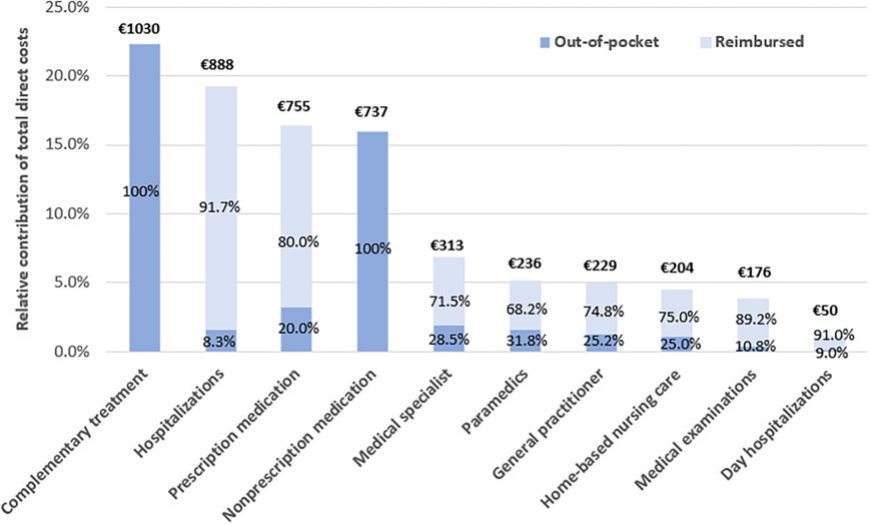
Relative contribution (%) of direct cost categories, further stratified between out-of-pocket and reimbursed costs, with mean cost (€) per cost category on top. Exact mean estimates with confidence intervals and median estimates with IQRs can be found in table 2

Total mean annual direct costs per patient amounted to €4618 (95% confidence interval: €4070–5152), of which 49.5% were out-of-pocket costs (table 2). Out-of-pocket costs were mainly explained by complementary treatment and non-prescription medication, which together accounted for €1766 (€1528–2024), or 38.2% of total direct costs (figure 1). The mean complementary treatment costs were largely driven by a minority of the patient population, whereas substantial non-prescription medication costs were reported by the majority (table 2).

**Table 2:** Mean and median annual direct and indirect costs (€)/cost category, Belgium, 2018

Cost category	Mean	BCa 95% CI lower	BCa 95% CI higher	Median cost (IQR)	% out-of-pocket
General practitioner visits	229.48	193.44	268.10	157.62 (0.00–315.24)	25.16
Medical specialist visits	313.46	261.39	375.61	236.28 (93.00–377.82)	28.51
Medical examinations	176.45	143.35	213.49	13.26 (0.00–234.50)	10.78
Home-based nursing care	204.11	107.38	323.96	0.00 (0.00–135.00)	24.96
Paramedic visits	235.65	158.41	315.98	0.00 (0.00–0.00)	31.80
Hospitalizations	887.55	612.42	1173.82	0.00 (0.00–0.00)	8.26
Day hospitalizations	50.00	34.81	65.04	0.00 (0.00–0.00)	9.00
Prescription medication	754.77	596.96	947.57	369.31 (51.25–830.58)	20.00
Non-prescription medication	736.62	638.24	867.32	828.12 (213.74–848.99)	100.00
Complementary treatments	1029.65	831.15	1254.32	300.00 (0.00–1838.14)	100.00
Total direct costs	4617.73	4069.66	5152.33	3152.78 (1731.95–6387.40)	49.53
Indirect costs	36 080.76	31 311.74	40 922.81	34 080.00 (0.00–68 160.00)	–

Bootstrap results based on 1000 iterations with 95% bias-corrected and accelerated (BCa) confidence intervals (CI). IQR, interquartile range.

Mean and median indirect costs amounted to €36 081 (€31 312–40 923) and €34 080 (IQR: €0–68 160), respectively. These costs were driven by the high number of patients (87 out of 187; 46.5%) who reported to be unemployed or absent from work for more than 1 year due to Lyme disease.

Extrapolation of the obtained results to the Belgian population, with an estimated PTLDS prevalence of 4199 patients, resulted in an estimated total annual disease burden of €19 389 848 for direct costs and €151 503 111 for indirect costs.

### Regression models

The regression model with total direct costs as the dependent variable yielded significant results for the covariates age category and income type (table 3). Patients of ≥60 years old and 40–59 years old incurred 54.8% (23.0–73.4%) and 41.6% (21.5–56.6%) less costs, respectively, compared to patients <40 years old. Patients receiving sickness/disability benefit or no income incurred 79.6% (34.7–139.4%) and 71.5% (9.9–167.6%) higher costs, respectively, compared to patients receiving an income from work. Gender, region, education level and time to diagnosis did not yield significant results.

**Table 3:** Regression with log-link function and gamma distribution with ‘total direct costs’ as dependent variable

Parameter	*B*	Standard error	Wald test, *P*-value	Percent change compared to reference (%)	95% Wald confidence interval	
					Lower (%)	Upper (%)
(Intercept)	8230	0.26	<0.001			
Gender						
Male	0.009	0.18	0.962	0.9	−29.4	44.2
Female	0^a^					
Age category						
≥60	−0.793	0.27	0.003	−54.8	−73.4	−23.0
40–59	−0.538	0.15	<0.001	−41.6	−56.6	−21.5
<40	0^a^					
Region						
Brussels	−0.302	0.52	0.559	−26.1	−73.1	103.4
Wallonia	0.194	0.20	0.338	21.5	−18.4	80.7
Flanders	0^a^					
Education						
Primary	−0.172	0.34	0.614	−15.8	−56.9	64.4
Secondary	0.282	0.20	0.159	32.6	−10.5	96.2
University college	0.234	0.18	0.196	26.3	−11.4	80.1
University	0^a^					
Income type						
Sickness or disability benefit	0.585	0.15	<0.001	79.6	34.7	139.4
No income	0.54	0.23	0.017	71.5	9.9	167.6
All other	0.165	0.21	0.422	17.9	−21.1	76.2
Work	0^a^					
Time to diagnosis						
≥10 years	−0.031	0.18	0.864	−3.1	−32.2	38.5
5–10 years	0.074	0.20	0.713	7.7	−27.4	59.8
1–5 years	−0.286	0.22	0.184	−24.9	−50.7	14.5
<1 year	0^a^					
(Scale)	0.597^b^	0.07				

Dependent variable: total direct costs. Intercept: €3753 (95% CI: €2251–6260).

a Reference value.

b Maximum likelihood estimate. Model: (intercept), gender, age category, region, education, income type, time to diagnosis.

The regression model with out-of-pocket costs as the dependent variable only yielded significant results for income type. Patients receiving sickness/disability benefit incurred 63.0% (15.7–129.6%) higher costs compared to patients receiving an income from work (Supplementary table S1). Similar results, with significant results for income type only, were obtained from the regression models with indirect costs and total costs as the dependent variables (Supplementary tables S2 and S3).

## Discussion

This study examined the direct and indirect costs of PTLDS-confirmed patients in Belgium. Based on an estimated population prevalence of 4199 patients, and direct and indirect costs of €4617 and 36 081 per patient, respectively, total annual societal costs were estimated as €170 892 959. Direct costs were high for out-of-pocket expenditures, largely due to the substantial contribution of both complementary therapies and non-prescribed medication.

Our estimation is considerably higher than the recently calculated societal cost of €5.6M in the first year after diagnosis in Belgium.^24^ This difference can largely be explained by differences in the patient population (Geebelen et al. included only a few patients with PTLDS), the population size estimation (Geebelen et al.’s^24^ estimation is incidence-based whereas our calculation is prevalence-based) and the methodological approach.

The cost of complementary therapies was previously assessed by van den Wijngaard et al.^22^ in the Netherlands, who identified a cost of €120 per patient with persisting symptoms based on complementary consultations. However, complementary therapy costs in our study were nine times higher. This difference might be, in part, explained by methods used. While both studies used self-reported questionnaires, the obtained data were thus subject to how participants interpreted questions, an issue also identified by van den Wijngaard et al.^22^ The question on complementary ‘therapies’ in this study might have been interpreted more broadly compared to the question by van den Wijngaard et al. on complementary ‘consultations’. Indeed, the Dutch study also reported an ‘other cost’ category, reinforcing this potential explanation.

Another noticeable result was the high mean period of 112.7 months (±9.4 years) from first symptoms to receiving a Lyme diagnosis in our study population. Physicians may not always think of Lyme disease when patients present with mild to more severe symptoms including fatigue, cognitive difficulties and musculoskeletal pain. Such a scenario may result in non-ideal treatment initiation and consequently persistent symptoms. Although the majority of patients return to their pre-morbid health, a subset of patients continue to report so-called subjective but long-term symptoms. It is not easy to diagnose and treat PTLDS at that moment, as universal guidelines for diagnosis and treatment are lacking.^19^ PTLDS remains subject to debate because of the poorly understood epidemiology and origins of the condition, and overlapping symptom profiles of comorbidities.^13^ Many PTLDS patients rely on complementary treatments in search of symptom relief. They explore multiple available treatments with no guaranteed success. Lantos et al.^38^ identified and reviewed more than 30 alternative treatments that are marketed to treat Lyme disease [e.g. nutritional therapy, (reactive) oxygen therapy, biological and pharmacological therapies], but failed to any supporting scientific evidence. Furthermore, several of the investigated therapies are potentially harmful.^38^ Patients who have a low locus of control about their health, who have hopeful/wishful thinking, and who are sceptical about conventional medicine tend to be attracted to alternative therapies in particular. Lantos et al.^38^ did not capture all possible complementary treatments; however, our focus was on the impact of out-of-pocket expenditure by patients, as our analyses showed that patients with sickness or disability benefit as income spent more. Thus, the severity of disease is likely associated with more despair. Moreover, these patients were at a higher risk of poverty.^39^

Importantly, patients under 40 were found to incur more direct costs compared to patients over 40 and 60. Our data show that this difference could be assigned to differences in reimbursed care-related costs. This might be explained by a shorter time since diagnosis in these younger adults, and hence still more healthcare seeking behaviour in the regular care circuit.

Caution is required when comparing cost-of-illness studies across countries, due to differences in scientific methodology, healthcare structure and social security systems. Our results in Belgium were compared with results in neighbouring countries by calculating the cost per capita (i.e. costs divided by the country’s population in a given year). We found that estimated direct costs were substantially higher in our study (direct cost per capita of 1.69) compared to Germany (0.31)^23^ and the Netherlands (0.53).^22^ The estimated costs for Germany were likely underestimated,^20^^,^^23^ while those of the Netherlands might have lacked accuracy, due to a short recall period (4 weeks) from which the entire year was extrapolated.^22^ However, our results might be overestimated, as the recall period of 6 months might have been too long. Patients have a good recall concerning hospitalization, whereas outpatient healthcare utilization recall is less reliable.^40^

This study had several limitations inherent to retrospective self-reporting over long periods. First, information on the number of PTLDS patients in Belgium was lacking, which was required to extrapolate the results from the individual patient level to population level. Thus, we used data from a study in the Netherlands^22^ as a proxy, while accounting for differences in EM and disseminated Lyme cases between the two countries.^36^^,^^37^ Second, Lyme-attributable costs related to emergency department visits were not included, because of a lack of data. These two limitations relate to missing information, which is interesting as the aim of cost-of-illness studies is to examine the socio-economic impact of diseases and identify data gaps and desirable refinements in national registration systems.^41^ Third, several respondents reported using a certain type of healthcare, but did not provide quantitative information. A mean cost value was used in these cases, which was based on the calculated costs of individuals who provided detailed information on health care resource use. Fourth, the convenience sampling method limited our ability to generalize the study results to the total PTLDS population in Belgium, for which sociodemographic and disease characteristics remain unknown to the best of our knowledge, probably due to existing controversies and a lack of guidelines.^19^ Our sample size consisted for 80% of women, which might still be an overrepresentation despite women are reported to show enduring symptoms compared to men.^42^ For comparison, women represented 67.7% of all in cases in a recent prospective study in Belgium.^43^ However, this may not have such an impact on representability and extrapolation as our analyses showed no cost differences between men and women. However, other characteristics may limit extrapolation.

Future research should, among other elements, focus on possibly relevant covariates, such as ethnicity or belief systems to predict Lyme-related direct medical costs and the use of complementary therapies in particular. It is of major importance to gain insight into which patients have a greater likelihood to experience difficulties in navigating the health care system, and to develop tailored answers on their call for medical help and support. In that regard it is also beneficial to analyze patterns of patients’ healthcare seeking behaviour.

## Conclusions

This study presented the first cost-of-illness analysis on PTLDS patients in Belgium. The results indicate high patient out-of-pocket expenses, especially with respect to unprescribed medication and complementary therapies. This phenomenon was particularly noticeable for patients with an income stemming from sickness or disability benefits. In conclusion, patients, especially those with high disease severity, encounter difficulties in finding appropriate care via conventional medical circuits.

Based on our study findings, the main implications are that more awareness should be created among policy makers about the health and economic consequences of PTLDS. There is also an urgent need for guidance on diagnosis and appropriate treatment.

## Ethical approval and consent to participate

The study was performed in accordance with the Declaration of Helsinki and approval to perform the study was obtained from the Ethics Committee of the University Hospital in Ghent (Belgium) [reference 2018/0933]. Informed consent was obtained from all participants. They were informed about the purpose of the study that participation in the study was voluntary, the data analysis was anonymous and they could withdraw from the study at any time.

## Supplementary Material

ckad045_Supplementary_Data

## Data Availability

The datasets generated and analyzed during this study are not publicly available due to privacy issues but are available from the corresponding author on reasonable request.

## References

[1] Steere AC , Strle F, Wormser GP, et al Lyme borreliosis. Nat Rev Dis Primers 2016;2:16090. 27976670 10.1038/nrdp.2016.90 PMC5539539

[2] Rizzoli A , Hauffe H, Carpi G, et al Lyme borreliosis in Europe. Euro Surveill 2011;16:19906. 21794218

[3] Sykes RA , Makiello P. An estimate of Lyme borreliosis incidence in Western Europe†. J Public Health (Oxf) 2017;39:74–81. 26966194 10.1093/pubmed/fdw017

[4] Perronne C. Lyme and associated tick-borne diseases: global challenges in the context of a public health threat. Front Cell Infect Microbiol 2014;4:74. 24918091 10.3389/fcimb.2014.00074 PMC4042490

[5] Vanthomme K , Bossuyt N, Boffin N, Van Casteren V. Incidence and management of presumption of Lyme borreliosis in Belgium: recent data from the sentinel network of general practitioners. Eur J Clin Microbiol Infect Dis 2012;31:2385–90. 22391757 10.1007/s10096-012-1580-3

[6] Editorial. Introducing EU-wide surveillance of Lyme neuroborreliosis. Lancet 2018;392:452. 10.1016/S0140-6736(18)31738-0 30129444

[7] Biesiada G , Czepiel J, Leśniak MR, et al Lyme disease: review. Arch Med Sci 2012;8:978–82. 23319969 10.5114/aoms.2012.30948 PMC3542482

[8] Coburn J , Leong J, Chaconas G. Illuminating the roles of the *Borrelia burgdorferi* adhesins. Trends Microbiol 2013;21:372–9. 23876218 10.1016/j.tim.2013.06.005 PMC3773214

[9] Jares TM , Mathiason MA, Kowalski TJ. Functional outcomes in patients with *Borrelia burgdorferi* reinfection. Ticks Tick Borne Dis 2014;5:58–62. 24215678 10.1016/j.ttbdis.2013.09.002

[10] Kullberg BJ , Vrijmoeth HD, van de Schoor F, Hovius JW. Lyme borreliosis: diagnosis and management. BMJ 2020;369:m1041. 32457042 10.1136/bmj.m1041

[11] Ali A , Vitulano L, Lee R, et al Experiences of patients identifying with chronic Lyme disease in the healthcare system: a qualitative study. BMC Fam Pract 2014;15:79. 24885888 10.1186/1471-2296-15-79 PMC4012507

[12] Rebman AW , Bechtold KT, Yang T, et al The clinical, symptom, and quality-of-life characterization of a well-defined group of patients with posttreatment Lyme disease syndrome. Front Med 2017;4:224. 10.3389/fmed.2017.00224 PMC5735370 29312942

[13] Rebman AW , Aucott JN. Post-treatment Lyme disease as a model for persistent symptoms in Lyme disease. Front Med 2020;7:57. 10.3389/fmed.2020.00057 PMC7052487 32161761

[14] Berndtson K. Review of evidence for immune evasion and persistent infection in Lyme disease. Int J Gen Med 2013;6:291–306. 23637552 10.2147/IJGM.S44114 PMC3636972

[15] Horowitz RI , Freeman PR. Precision medicine: the role of the MSIDS model in defining, diagnosing, and treating chronic Lyme disease/post treatment Lyme disease syndrome and other chronic illness: part 2. Healthcare (Basel) 2018;6:129. 30400667 10.3390/healthcare6040129 PMC6316761

[16] Jacek E , Fallon BA, Chandra A, et al Increased IFNα activity and differential antibody response in patients with a history of Lyme disease and persistent cognitive deficits. J Neuroimmunol 2013;255:85–91. 23141748 10.1016/j.jneuroim.2012.10.011 PMC3557545

[17] Stricker RB , Johnson L. *Borrelia burgdorferi* aggrecanase activity: more evidence for persistent infection in Lyme disease. Front Cell Infect Microbiol 2013;3:40. 23967405 10.3389/fcimb.2013.00040 PMC3743303

[18] US Department of Health and Human Services. Tick-Borne Disease Working Group. 2018 Report to Congress. 2018. Available at: https://www.hhs.gov/sites/default/files/tbdwg-report-to-congress-2018.pdf?fbclid=IwAR1u5BTMGMUMCIdMOfudGAqChW1psJL421l76FXWWn4u-YYh2edmRR0LHyw (8 September 2022, date last accessed).

[19] Maksimyan S , Syed MS, Soti V. Post-treatment Lyme disease syndrome: need for diagnosis and treatment. Cureus 2021;13:e18703. 34659931 10.7759/cureus.18703 PMC8507427

[20] Mac S , da Silva SR, Sander B. The economic burden of Lyme disease and the cost-effectiveness of Lyme disease interventions: a scoping review. PLoS One 2019;14:e0210280. 30608986 10.1371/journal.pone.0210280 PMC6319811

[21] Henningsson AJ , Malmvall BE, Ernerudh J, et al Neuroborreliosis–an epidemiological, clinical and healthcare cost study from an endemic area in the south-east of Sweden. Clin Microbiol Infect 2010;16:1245–51. 19793326 10.1111/j.1469-0691.2009.03059.x

[22] van den Wijngaard CC , Hofhuis A, Wong A, et al The cost of Lyme borreliosis. Eur J Public Health 2017;27:538–47. 28444236 10.1093/eurpub/ckw269

[23] Lohr B , Muller I, Mai M, et al Epidemiology and cost of hospital care for Lyme borreliosis in Germany: lessons from a health care utilization database analysis. Ticks Tick Borne Dis 2015;6:56–62. 25448420 10.1016/j.ttbdis.2014.09.004

[24] Geebelen L , Devleesschauwer B, Lernout T, et al Lyme borreliosis in Belgium: a cost-of-illness analysis. BMC Public Health 2022;22:2194. 36443755 10.1186/s12889-022-14380-6 PMC9703731

[25] Rice DP , Hodgson TA, Sinsheimer P, et al The economic costs of the health effects of smoking, 1984. Milbank Q 1986;64:489–547. 3102916

[26] Moore TJ , Caulkins JP. How cost-of-illness studies can be made more useful for illicit drug policy analysis. Appl Health Econ Health Policy 2006;5:75–85. 16872249 10.2165/00148365-200605020-00002

[27] Drummond M , Sculpher M, Claxton K, et al Methods for the economic evaluation of health care programmes, 4th edn. Oxford: Oxford University Press, 2015.

[28] Nomenclature database. 2018. Available at: https://ondpanon.riziv.fgov.be/Nomen/nl/search (15 January 2019, date last accessed).

[29] Kohn L , Obyn C, Adriaenssens J, et al Model for the organization and reimbursement of psychological and orthopedagogical care in Belgium. Health Services Research (HSR). KCE Reports 265. D/2016/10.273/34. Brussels, 2016.

[30] FPS Health, NIHDI. National database medical diagnosis/care and cost. 2017.

[31] Van de Sande S , Swartenbroekx N, Van de Voorde C, et al Evolution of day-care: impact of financing and regulation. Health Services Research (HSR). KCE Reports 192A. D/2012/10.273/89. Brussels, 2012.

[32] Gecommentarieerd geneesmiddelenrepertorium. 2018. Available at: http://www.bcfi.be/nl/chapters (18 August 2022, date last accessed).

[33] Crommelynck A , Degraeve K, Lefèbvre D. De organisatie en financiering van de ziekenhuizen [Organization and financing of hospitals]. Christelijke Mutualiteit 2013;253:1–44.

[34] Verlinden H. Absenteïsme in 2018 (absenteism in 2018). Securex Report. Brussels: Frank Vander Sijpe, Securex Corporate EESV, 2019. Available at: https://www.securex.be/nl (12 January 2020, date last accessed).

[35] Hankivsky O , Friesen J, Varcoe C, et al Expanding economic costing in health care: values, gender and diversity. Can Public Policy Anal Polit 2004;30:257–82.

[36] Geebelen L , Van Cauteren D, Devleesschauwer B, et al Combining primary care surveillance and a meta-analysis to estimate the incidence of the clinical manifestations of Lyme borreliosis in Belgium, 2015–2017. Ticks Tick Borne Dis 2019;10:8. 10.1016/j.ttbdis.2018.12.007 30772196

[37] STATBEL. Structuur van de bevolking [Structure of the population]: STATBEL. 2018 Available at: https://statbel.fgov.be/nl/themas/bevolking/structuur-van-de-bevolking (12 January 2020, date last accessed).

[38] Lantos PM , Shapiro ED, Auwaerter PG, et al Unorthodox alternative therapies marketed to treat Lyme disease. Clin Infect Dis 2015;60:1776–82. 25852124 10.1093/cid/civ186 PMC4490322

[39] Capéau B , Cherchye L, Decanq K, et al In: Silber J, Gan R, editors. Well-Being in Belgium: Beyond Happiness and Income. Cham, Switzerland: Springer Nature, 2015: 33–42.

[40] Petrou S , Murray L, Cooper P, Davidson LL. The accuracy of self-reported healthcare resource utilization in health economic studies. Int J Technol Assess Health Care 2002;18:705–10. 12391960 10.1017/s026646230200051x

[41] Single E. Why we should still estimate the costs of substance abuse even if we needn't pay undue attention to the bottom line. Drug Alcohol Rev 2009;28:117–21. 19320695 10.1111/j.1465-3362.2008.00040.x

[42] Wormser GP , Shapiro ED. Implications of gender in chronic Lyme disease. J Womens Health (Larchmt) 2009;18:831–4. 19514824 10.1089/jwh.2008.1193 PMC2913779

[43] Geebelen L , Lernout T, Devleesschauwer B, et al Non-specific symptoms and post-treatment Lyme disease syndrome in patients with Lyme borreliosis: a prospective cohort study in Belgium (2016-2020). BMC Infect Dis 2022;22:756. 36171561 10.1186/s12879-022-07686-8 PMC9518937

