# Supplementary material for: Cost of illness in patients with post-treatment Lyme disease syndrome in Belgium
Source: Eur J Public Health. 2023 Mar 27;33(4):668–74. doi: 10.1093/eurpub/ckad045 (PMC10393486; doi:10.1093/eurpub/ckad045)
Supplement: ckad045_Supplementary_Data [file ckad045_supplementary_data.docx]

# Supplementary material paper COI PTLDS

**Table S1. Regression with log-link function and gamma distribution with ‘out-of-pocket expenditures’ as dependent variable.**

| Parameter |  | B | Std. Error | Wald Test, p-value | Percent change compared to reference | 95% Wald Confidence Interval | |
| --- | --- | --- | --- | --- | --- | --- | --- |
|  |  |  |  |  |  | Lower | Upper |
| (Intercept) |  | 7.613 | 0.26 |  |  |  |  |
| Gender | Male | 0.022 | 0.20 | 0.912 | 2.3% | -31.2% | 52.0% |
|  | Female | 0^a^ |  |  | 1 |  |  |
| Age category | ≥60 | -0.445 | 0.27 | 0.096 | -35.9% | -62.1% | 8.2% |
|  | 40-59 | -0.367 | 0.17 | 0.029 | -30.7% | -50.2% | -3.6% |
|  | <40 | 0^a^ |  |  | 1 |  |  |
| Region | Brussels | -0.385 | 0.53 | 0.47 | -31.9% | -76.0% | 93.4% |
|  | Wallonia | 0.017 | 0.17 | 0.921 | 1.7% | -27.5% | 42.6% |
|  | Flanders | 0^a^ |  |  | 1 |  |  |
| Education | Primary | -0.192 | 0.40 | 0.628 | -17.5% | -62.1% | 79.5% |
|  | Secondary | 0.205 | 0.21 | 0.334 | 22.8% | -19.0% | 86.2% |
|  | University college | 0.171 | 0.18 | 0.348 | 17.8% | -17.0% | 69.8% |
|  | University | 0^a^ |  |  | 1 |  |  |
| Income Type | Sickness and disability benefit | 0.489 | 0.17 | 0.005 | 63.0% | 15.7% | 129.6% |
|  | No income | 0.163 | 0.23 | 0.482 | 17.7% | -25.3% | 85.7% |
|  | All other | -0.083 | 0.22 | 0.699 | -8.0% | -39.6% | 40.3% |
|  | Work | 0^a^ |  |  | 1 |  |  |
| Time to diagnosis | ≥10 year | -0.015 | 0.19 | 0.935 | -1.5% | -31.5% | 41.7% |
|  | 5-10 year | 0.033 | 0.22 | 0.881 | 3.4% | -32.9% | 59.3% |
|  | 1-5 year | -0.168 | 0.25 | 0.502 | -15.5% | -48.2% | 38.0% |
|  | <1 year | 0^a^ |  |  | 1 |  |  |
| (Scale) |  | 0.738^b^ | 0.08 |  |  |  |  |
| Dependent Variable: Out-of-pocket expenditures. Intercept: €2024 (95% CI: €1213-€3378). ^a^Reference value. ^b^Maximum likelihood estimate. Model: (Intercept), Gender, Age category, Region, Education, Income Type, Time to diagnosis. | | | | | | | |

**Table S2. Regression with log-link function and Tweedie distribution with ‘indirect costs’ as dependent variable.**

| Parameter |  | B | Std. Error | Wald Test, p-value | Percent change compared to reference | 95% Wald Confidence Interval | |
| --- | --- | --- | --- | --- | --- | --- | --- |
|  |  |  |  |  |  | Lower | Upper |
| (Intercept) |  | 9.115 | 0.42 |  |  |  |  |
| Gender | Male | 0.075 | 0.15 | 0.608 | 7.8% | -19.0% | 43.4% |
|  | Female | 0^a^ |  |  |  |  |  |
| Age category | ≥60 | -0.598 | 0.58 | 0.3 | -45.0% | -82.2% | 70.2% |
|  | 40-59 | -0.15 | 0.17 | 0.384 | -13.9% | -38.6% | 20.7% |
|  | <40 | 0^a^ |  |  |  |  |  |
| Region | Brussels | 0.118 | 0.44 | 0.79 | 12.5% | -52.8% | 168.4% |
|  | Wallonia | -0.61 | 0.15 | 0.679 | -5.9% | -29.4% | 25.4% |
|  | Flanders | 0^a^ |  |  |  |  |  |
| Education | Primary | -0.211 | 0.34 | 0.54 | -19.0% | -58.7% | 58.9% |
|  | Secundary | -0.069 | 0.20 | 0.732 | -6.6% | -37.1% | 38.5% |
|  | University college | -0.039 | 0.19 | 0.839 | -3.8% | -33.8% | 39.8% |
|  | University | 0^a^ |  |  |  |  |  |
| Income Type | Sickness and disability benefit | 1.724 | 0.25 | <0.001 | 460.8% | 241.6% | 820.6% |
|  | No income | 1.484 | 0.32 | <0.001 | 341.2% | 133.6% | 733.6% |
|  | All other | 1.071 | 0.35 | 0.003 | 191.8% | 45.7% | 484.5% |
|  | Work | 0^a^ |  |  |  |  |  |
| Time to diagnosis | ≥10 year | 0.393 | 0.25 | 0.121 | 48.1% | -9.9% | 143.5% |
|  | 5-10 year | 0.39 | 0.24 | 0.101 | 47.7% | -7.3% | 135.2% |
|  | 1-5 year | 0.155 | 0.23 | 0.508 | 16.7% | -26.2% | 84.6% |
|  | <1 year | 0^a^ |  |  |  |  |  |
| (Scale) |  | 106.146^b^ | 90.97 |  |  |  |  |
| Dependent Variable: Indirect costs. Intercept: €9091 (95% CI: €3954-€20,904). ^a^Reference value. ^b^Maximum likelihood estimate. Model: (Intercept), Gender, Age category, Region, Education, Income Type, Time to diagnosis. | | | | | | | |

**Table S3. Regression with log-link function and gamma distribution with ‘total costs’ as dependent variable.**

| Parameter |  | B | Std. Error | Wald Test, p-value | Percent change compared to reference | 95% Wald Confidence Interval | |
| --- | --- | --- | --- | --- | --- | --- | --- |
|  |  |  |  |  |  | Lower | Upper |
| (Intercept) |  | 9.361 | 0.38 |  |  |  |  |
| Gender | Male | 0.001 | 0.18 | 0.998 | 0.1% | -29.8% | 42.6% |
|  | Female | 0^a^ |  |  |  |  |  |
| Age category | ≥60 | -0.372 | 0.61 | 0.541 | -31.0% | -79.0% | 126.7% |
|  | 40-59 | -0.218 | 0.19 | 0.254 | -19.4% | -44.8% | 17.0% |
|  | <40 | 0^a^ |  |  |  |  |  |
| Region | Brussels | -0.088 | 0.46 | 0.848 | -8.5% | -73.0% | 126.6% |
|  | Wallonia | -0.09 | 0.20 | 0.656 | -8.6% | -38.3% | 35.6% |
|  | Flanders | 0^a^ |  |  |  |  |  |
| Education | Primary | -0.244 | 0.43 | 0.572 | -21.7% | -66.4% | 82.7% |
|  | Secundary | -0.043 | 0.22 | 0.849 | -4.2% | -38.3% | 48.9% |
|  | University college | -0.009 | 0.23 | 0.97 | -0.9% | -37.0% | 56.0% |
|  | University | 0^a^ |  |  |  |  |  |
| Income Type | Sickness and disability benefit | 1.594 | 0.20 | <0.001 | 392.3% | 235.9% | 621.7% |
|  | No income | 1.349 | 0.30 | <0.001 | 285.3% | 114.2% | 593.0% |
|  | All other | 0.808 | 0.34 | 0.016 | 124.4% | 16.0% | 334.1% |
|  | Work | 0^a^ |  |  |  |  |  |
| Time to diagnosis | ≥10 year | 0.506 | 0.27 | 0.061 | 65.8% | -2.2% | 181.2% |
|  | 5-10 year | 0.44 | 0.26 | 0.93 | 55.3% | -7.1% | 159.7% |
|  | 1-5 year | 0.138 | 0.26 | 0.597 | 14.8% | -31.2% | 91.7% |
|  | <1 year | 0^a^ |  |  |  |  |  |
| (Scale) |  | 0.997^b^ | 0.11 |  |  |  |  |
| Dependent Variable: Total costs (direct plus indirect). Intercept: €11,624 (95% CI: €5527-€24,446). ^a^Reference value. ^b^Maximum likelihood estimate. Model: (Intercept), Gender, Age category, Region, Education, Income Type, Time to diagnosis. | | | | | | | |
